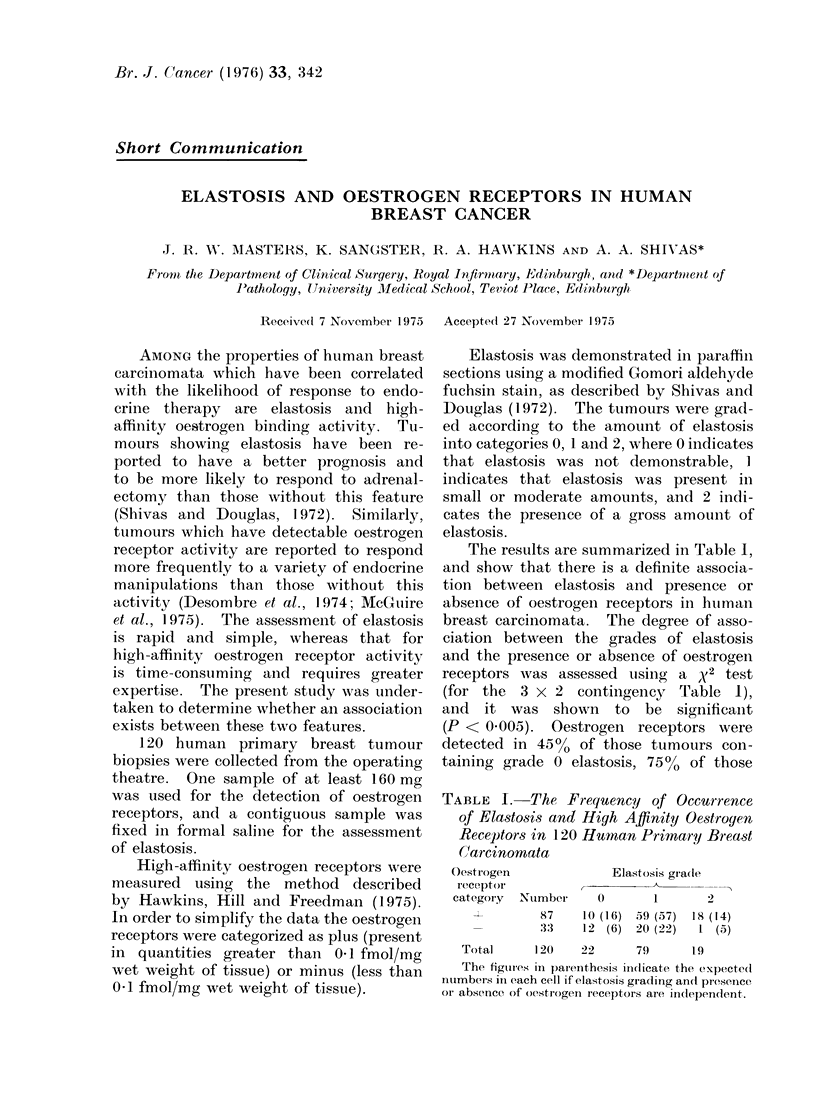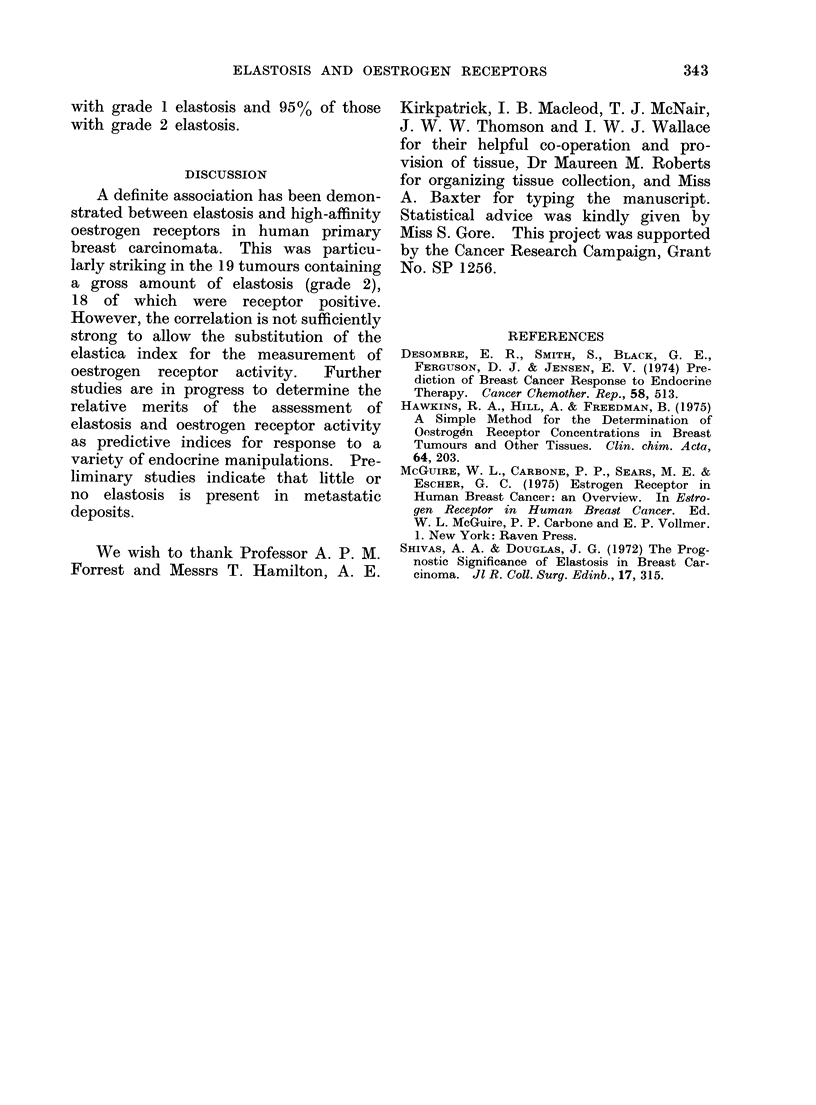# Elastosis and oestrogen receptors in human breast cancer.

**DOI:** 10.1038/bjc.1976.49

**Published:** 1976-03

**Authors:** J. R. Masters, K. Sangster, R. A. Hawkins, A. A. Shivas


*Br. J. Cancer* (1976) **33**, 342

***Short Communication***

### ELASTOSIS AND OESTROGEN RECEPTORS IN HUMAN BREAST CANCER

J. R. W. MASTERS, K. SANGSTER, R. A. HAWKINS AND A. A. SHIVAS*

*From the Department of Clinical Surgery, Royal Infirmary, Edinburgh, and *Department of Pathology, University Medical School, Teviot Place, Edinburgh*

Received 7 November 1975 Accepted 27 November 1975

AMONG the properties of human breast carcinomata which have been correlated with the likelihood of response to endocrine therapy are elastosis and high-affinity oestrogen binding activity. Tumours showing elastosis have been reported to have a better prognosis and to be more likely to respond to adrenalectomy than those without this feature (Shivas and Douglas, 1972). Similarly, tumours which have detectable oestrogen receptor activity are reported to respond more frequently to a variety of endocrine manipulations than those without this activity (Desombre *et al.*, 1974; McGuire *et al.*, 1975). The assessment of elastosis is rapid and simple, whereas that for high-affinity oestrogen receptor activity is time-consuming and requires greater expertise. The present study was undertaken to determine whether an association exists between these two features.

120 human primary breast tumour biopsies were collected from the operating theatre. One sample of at least 160 mg was used for the detection of oestrogen receptors, and a contiguous sample was fixed in formal saline for the assessment of elastosis.

High-affinity oestrogen receptors were measured using the method described by Hawkins, Hill and Freedman (1975). In order to simplify the data the oestrogen receptors were categorized as plus (present in quantities greater than 0·1 fmol/mg wet weight of tissue) or minus (less than 0·1 fmol/mg wet weight of tissue).

Elastosis was demonstrated in paraffin sections using a modified Gomori aldehyde fuchsin stain, as described by Shivas and Douglas (1972). The tumours were graded according to the amount of elastosis into categories 0, 1 and 2, where 0 indicates that elastosis was not demonstrable, 1 indicates that elastosis was present in small or moderate amounts, and 2 indicates the presence of a gross amount of elastosis.

The results are summarized in Table I, and show that there is a definite association between elastosis and presence or absence of oestrogen receptors in human breast carcinomata. The degree of association between the grades of elastosis and the presence or absence of oestrogen receptors was assessed using a $\chi^2$ test (for the $3 \times 2$ contingency Table I), and it was shown to be significant ($P < 0{\cdot}005$). Oestrogen receptors were detected in 45% of those tumours containing grade 0 elastosis, 75% of those

TABLE I.—*The Frequency of Occurrence of Elastosis and High Affinity Oestrogen Receptors in* 120 *Human Primary Breast Carcinomata*

| Oestrogen receptor category | Number | Elastosis grade | | |
|---|---|---|---|---|
| | | 0 | 1 | 2 |
| + | 87 | 10 (16) | 59 (57) | 18 (14) |
| − | 33 | 12 (6) | 20 (22) | 1 (5) |
| Total | 120 | 22 | 79 | 19 |

The figures in parenthesis indicate the expected numbers in each cell if elastosis grading and presence or absence of oestrogen receptors are independent.

with grade 1 elastosis and 95% of those with grade 2 elastosis.

### DISCUSSION

A definite association has been demonstrated between elastosis and high-affinity oestrogen receptors in human primary breast carcinomata. This was particularly striking in the 19 tumours containing a gross amount of elastosis (grade 2), 18 of which were receptor positive. However, the correlation is not sufficiently strong to allow the substitution of the elastica index for the measurement of oestrogen receptor activity. Further studies are in progress to determine the relative merits of the assessment of elastosis and oestrogen receptor activity as predictive indices for response to a variety of endocrine manipulations. Preliminary studies indicate that little or no elastosis is present in metastatic deposits.

We wish to thank Professor A. P. M. Forrest and Messrs T. Hamilton, A. E. Kirkpatrick, I. B. Macleod, T. J. McNair, J. W. W. Thomson and I. W. J. Wallace for their helpful co-operation and provision of tissue, Dr Maureen M. Roberts for organizing tissue collection, and Miss A. Baxter for typing the manuscript. Statistical advice was kindly given by Miss S. Gore. This project was supported by the Cancer Research Campaign, Grant No. SP 1256.

### REFERENCES

DESOMBRE, E. R., SMITH, S., BLACK, G. E., FERGUSON, D. J. & JENSEN, E. V. (1974) Prediction of Breast Cancer Response to Endocrine Therapy. *Cancer Chemother. Rep.*, **58**, 513.

HAWKINS, R. A., HILL, A. & FREEDMAN, B. (1975) A Simple Method for the Determination of Oestrogen Receptor Concentrations in Breast Tumours and Other Tissues. *Clin. chim. Acta*, **64**, 203.

MCGUIRE, W. L., CARBONE, P. P., SEARS, M. E. & ESCHER, G. C. (1975) Estrogen Receptor in Human Breast Cancer: an Overview. In *Estrogen Receptor in Human Breast Cancer*. Ed. W. L. McGuire, P. P. Carbone and E. P. Vollmer. 1. New York: Raven Press.

SHIVAS, A. A. & DOUGLAS, J. G. (1972) The Prognostic Significance of Elastosis in Breast Carcinoma. *Jl R. Coll. Surg. Edinb.*, **17**, 315.